# Bausteine zu einer Oral History der Wissenschaftsgeschichte Auf der Suche: von der Biologie und der Philosophie zur Wissenschaftsgeschichte. Interview mit Soraya de Chadarevian

**DOI:** 10.1002/bewi.70001

**Published:** 2025-09-17

**Authors:** Soraya de Chadarevian, Mathias Grote, Anke te Heesen

**Affiliations:** ^1^ Department of History University of California, Los Angeles 315 Portola Plaza, 6265 Bunche Hall, Box 951473 Los Angeles CA 90095‐1473 USA; ^2^ Historisches Institut Universität Greifswald Domstr. 9a 17489 Greifswald Germany; ^3^ Institut für Geschichtswissenschaften Humboldt‐Universität zu Berlin Unter den Linden 6 10099 Berlin Germany

## Abstract

Wie kann man einen historischen Blick auf das eigene Fach werfen? Diese Frage ist nicht einfach zu beantworten – will man einerseits nicht in einer Nabelschau und Hagiographie enden, andererseits aber auch keinen umfassenden Entwurf einer zukünftigen Historiographie vorlegen. Die hier in loser Folge publizierten Interviews mit bekannten Protagonist:innen der Berliner Wissenschaftsgeschichte von ca. 1970–1990 in West und Ost rücken die Geschichte des Faches deshalb in einem bestimmten Milieu in den Fokus und versuchen, die Historiographie jenseits einer Institutionen‐ oder Theoriegeschichte voranzutreiben. Welche Motivationen oder Probleme bewegten einzelne Wissenschaftler:innen, sich der Geschichte ihres Faches zu widmen oder sich etwa aus der Soziologie oder Philosophie in die Wissenschaftsgeschichte zu bewegen? Welche Ausbildungspraktiken existierten in diesem heterogenen, zwischen den Disziplinen angesiedelten Feld, welche Anregungen bezog man aus welchen Kontexten? Wie war Lehre strukturiert und welche Netzwerke bildeten sich mit der Zeit? Kurz: Mit welchem Interesse kam man zur Wissenschaftsgeschichte und was wurde daraus?

Die Auswahl der Interviewees erfolgt ohne Anspruch auf Vollständigkeit oder Proporz; der Fragenkatalog der Interviews richtet sich individuell nach den Biographien und dem Werk und entfaltet sich oft spontan im Gespräch. Die Interviews wurden digital aufgezeichnet, transkribiert, der Schriftsprache angepasst, gegebenenfalls gekürzt, annotiert und von den Interviewees authentifiziert.

Wir beabsichtigen mit dieser Serie von Interviews zunächst die Dokumentation rezenter Geschichte durch eine Oral History, die subjektive Wahrnehmungen und persönliche Erlebnisse einschließt. Auf diese Weise werden Segmente einer größtenteils ungeschriebenen Geschichte anhand von Biographien erfahrbar und damit auch einer weiteren kritischen Bearbeitung und Integration in ein Gesamtbild zugänglich. Da uns im Zuge der jeweiligen Vorbereitung und Durchführung, Transkription und Abstimmung der Interviews daran gelegen war, aus Sicht der Akteur:innen wichtige Sammelbände und Aufsätze, Monographien oder auch „graue Literatur“ zu erfassen, wird nebenbei eine kommentierte Bibliographie zur Geschichte der Wissenschaftsgeschichte entstehen. Unsere Hoffnung besteht darin, mittels dieser Sammlung mit Berlin einen fruchtbaren Raum und mit den siebziger und achtziger Jahren eine produktive Zeit des Faches jenseits von Reminiszenz oder Nostalgie zu erkunden – nicht zuletzt auch, um den Blick für gegenwärtige Herausforderungen des Faches zu schärfen.

**Anke te Heesen (AtH)**: Liebe Soraya, am besten wir beginnen bei Deiner Ausbildung und den ersten Schritten in die Wissenschaftsgeschichte. Du hast zunächst Biologie studiert und abgeschlossen. Aber wie bist Du von dort zur Wissenschaftsgeschichte gekommen?

**Soraya de Chadarevian (SdC)**: Das stimmt. Biologie ist das einzige Fach, das ich systematisch studiert habe, und es hat mir eine feste Grundlage gegeben für fast alles, oder jedenfalls vieles, was ich nachher unternommen habe.

Ich habe Biologie in Freiburg studiert, mit einem DAAD‐Stipendium, das mich von Italien nach Deutschland gebracht hat. Es war mir anfänglich nicht so klar, aber es wurde dann doch schnell deutlich, was für ein exzellenter Fachbereich Freiburg war. Das Studium der Biologie war dort einige Jahre zuvor grundlegend reformiert worden. Carsten Bresch^1^ hatte ein neues Institut für Molekularbiologie eingerichtet – das Institut für Biologie III. Bresch war ein Schüler von Max Delbrück,^2^ einem *pioneer* der Molekularbiologie. Bevor er nach Freiburg kam, hatte er zusammen mit Delbrück das neue Institut für Genetik an der Universität Köln gegründet, das erste molekularbiologische Programm Deutschlands. Auch in Freiburg haben wir von diesem *re‐thinking der Biologie* profitiert – eben weg von Zoologie, Botanik und Systematik als Grundpfeilern des Fachs, hin zu einem stark naturwissenschaftlichen und eben auch molekular‐ und zellbiologisch ausgerichteten Studium. Und wir hatten für alle Fächer wirklich ausgezeichnete Professoren.

Wir mussten auch einen Kurs belegen, der hieß, glaube ich, „Wissenschaftstheoretische und ethische Probleme der Biologie“. Ich habe ihn mindestens zwei Mal belegt, da er von verschiedenen Professoren unterrichtet wurde, die das Thema ganz unterschiedlich angegangen sind. Grundsätzlich handelte es sich um eine Reflexion über die Grundlagen und Methoden der Biologie. Die Fragen, die wir damals diskutierten, waren zum Beispiel der Begriff der Teleonomie im Gegensatz zur Teleologie oder wie man, im Lichte der Molekularbiologie, Fragen der Evolution neu beantworten kann. Das ging auf Jacques Monods Buch zurück, das ich übrigens als Abiturabschiedsbuch von der Schule geschenkt bekam.^3^ Aber auch Fragen der Soziobiologie und der evolutionären Erkenntnistheorie waren damals akut. Mir schienen das schwierige Debatten, auf die es keine klare Antworten gab. Da habe ich oft gedacht, wenn man hier irgendwie weiterkommen will, dann braucht man mehr philosophisches Grundwissen … Jedenfalls war die Frage „Was machen wir hier eigentlich und wo steht die Biologie heute?“ eingebaut in das Studium. Auch das neue Lehrbuch zur Genetik von Carsten Bresch zum Beispiel war ein Versuch, den Stoff neu darzustellen, das heißt nicht nur Fakten zu vermitteln, sondern auch zu beschreiben, wie Wissen experimentell gewonnen wird.^4^ Ich glaube, das alles war sehr wichtig für mein späteres Interesse an der Wissenschaftsgeschichte. Eine andere wichtige Erfahrung war meine Diplom‐Arbeit, die ich in einem Labor Bologna geschrieben habe. In der Arbeit ging es um den Prozess der Energiegewinnung in der Zelle, für den es damals verschiedene Modelle gab. Darüber hinaus aber war die Arbeit praktisch eine Fallstudie in einer Soziologie der Wissenschaften, auch wenn ich mir darüber teilweise erst später klar geworden bin. Von Problemen der Replikation [von Experimenten], dem *experimenter's regress*, zur Bedeutung von *skills* und *tacit knowledge* im Experimentieren und am Ende ein Fall eines wissenschaftlichen *frauds –* all das erlebte ich *first‐hand* in meiner Diplomarbeit. Das war natürlich sehr lehrreich. Auch habe ich in meiner Zeit im Labor zum Beispiel Thomas Kuhn gelesen. Seine Begriffe von Paradigmen und normaler Wissenschaft haben mir sehr eingeleuchtet. Aus all diesen Gründen glaube ich wirklich, dass diese Jahre sehr wichtig waren, auch wenn mich mein Weg dann erst einmal in die Philosophie und erst später zur Wissenschaftsgeschichte gebracht hat.

**Mathias Grote (MG)**: Bevor wir zur Philosophie kommen, nochmal zur Biologie und den verschiedenen Instituten in Freiburg, zu den reflexiven Fragen: Hast Du das für Dich als Spannung wahrgenommen, einerseits diese reflexiven, philosophischen Fragen kennenzulernen und zu diskutieren, aber andererseits auch praktische Laborarbeit zu machen? Denn die Publikation, die offensichtlich aus deiner Diplom‐Arbeit mit entstanden ist, sieht ja aus wie eine ganz reguläre, experimentelle Arbeit, für die Du offensichtlich an der Laborbank gestanden bist.^5^

**SdC**: Ich habe das nicht als eine Spannung empfunden. Ich habe gern im Labor gearbeitet, und was es zusammengehalten hat, war die Frage, was Biologie ist. Trotzdem glaube ich, dass mein Weg ein wenig ungewöhnlich war. Also, erstmal sollte ich sagen, ich habe auf Biologie‐Diplom studiert. Wir fingen mit etwa 80 Leuten an und haben die ganze Zeit gehört, wie schlecht die Berufsaussichten sind, und so weiter. Als Antwort darauf sind praktisch alle auf Lehramt umgestiegen, außer sechs oder acht von uns. Also eine ganz kleine Gruppe, die wirklich Diplom‐Biologie studiert hat. Und wir brauchten für den Diplom‐Abschluss ein nicht‐biologisches Nebenfach. Die meisten haben Chemie oder ein anderes naturwissenschaftliches Fach gewählt, aber ich habe philosophische Anthropologie gemacht. Das war für mich okay, weil ich sowieso immer breit interessiert war und auch Biologie studiert habe, weil ich dachte, das ist ein Fach, wo ich meine breiten Interessen noch irgendwie einbringen kann.

Und ich bekam Unterstützung. Zum Beispiel war es Rainer Hertel,^6^ der meine Diplom‐Arbeit mitbetreut hat und bei dem ich danach noch ein Jahr im Labor gearbeitet habe, der mich ermutigt hat, in der Philosophie zu promovieren. Er sagte: „Danach hast Du beides. Das ist die Zukunft und Dir steht die Welt offen.“ Hertel war überzeugt, dass man Philosophie brauchte, um kritisch und sozusagen mit einem offenen *mind* Wissenschaft zu betreiben.

**AtH**: Das heißt, ein solches Vorbild wie Hertel spielte eine wichtige Rolle für Dich, er war philosophisch interessiert und förderte Dich auch in dieser Hinsicht.

**SdC**: Ja, aber ich habe nie in oder mit Vorbildern gedacht. Es gab einige Menschen, die für mich wichtig waren und Rainer Hertel gehört zweifellos dazu. Er war sehr jung Professor geworden, hat über Auxine, über die Hormone der Pflanzen, gearbeitet und ist übrigens auch derjenige, der mir später das Sachs‐Darwin‐Thema nahegelegt hat, mein erstes Forschungsprojekt in der Wissenschaftsgeschichte.^7^ Wir haben verschiedene Themen diskutiert, und darunter eben auch dieses – es ging ja praktisch um die Vorgeschichte seiner Forschung. Ich habe auch mit ihm weiter Kontakt gehalten.

**AtH**: Und dann bist Du in die Philosophie gewechselt.

**SdC**: Dann bin ich in die Philosophie gegangen. Übrigens waren diese Übergänge für mich nie ganz einfach, es gab immer auch die Möglichkeit, ganz was anderes zu machen. Dazu sollte ich vielleicht erklären: Ich hatte das DAAD‐Stipendium, um in Deutschland zu studieren. In Italien aber hatte man mit seinem Uni‐Abschluss – und mein Diplom wurde mir wegen meiner Zeit in Bologna auch als italienischer Abschluss anerkannt – schon einen Doktortitel. Ich war also schon *dottoressa in scienze biologiche*. Es gab damals noch kein Promotionsstudium im italienischen System, und ich habe mich ja noch am italienischen System orientiert. Ich wäre auch gerne weiter in Bologna geblieben, aber das bedeutete, für Jahre unbezahlt zu arbeiten und das ging in meinem Fall nicht. Im Gegensatz dazu wurde mir in Freiburg angeboten, weiter im Labor zu arbeiten und auch eine Hiwi‐Stelle zu bekommen. Das habe ich dann ein Jahr gemacht. Aber dann hatte ich die Idee, wenn ich eine Promotion mache – was ich, wie gesagt, nicht unbedingt vorhatte – dann wollte ich was Neues machen. Viele haben mir damals gesagt: „Du bist doch jetzt schon im Labor, du hast doch praktisch deine Dissertation schon angefangen. Wir haben alle unsere Zweifel gehabt, aber dann haben wir doch weitergemacht.“ Aber ich blieb bei meiner Idee und habe dann verschiedene Programme und Möglichkeiten erwogen – damals noch durchaus mit der Idee, irgendwann mal wieder in die experimentelle Forschung zurückzugehen.

**AtH**: Wie kam es dann zum Promotionsstudium in Konstanz?

**SdC**: Konstanz hatte ein Programm, in dem man auch mit einem Abschluss in den Naturwissenschaften in der Philosophie promovieren konnte. Das war *sehr* ungewöhnlich, aber kam mir genau entgegen. Andere Möglichkeiten, die ich erwog, zum Beispiel bei Jean Piaget^8^ oder Claude Lévi‐Strauss^9^ zu promovieren – diese Ideen kamen von meiner Beschäftigung mit der philosophischen Anthropologie –, die verlangten alle, dass ich von vorn anfangen und also ein zweites Grundstudium machen sollte. Doch das wollte ich nicht, ich wollte ja nicht die Disziplin wechseln, sondern einfach nur weiterdenken. Das also hat mich sehr für Konstanz eingenommen. Es stellte sich heraus, dass ich zu meinem Beginn dort 1979 die einzige Studentin in diesem Programm war, aber das war nicht wirklich ein Problem. Obwohl ich schon im Promotionsstudium war, habe ich viele Kurse belegt, weil ich das Gefühl hatte, dass ich viel nachzuholen hatte. Aber ein bisschen eine Basis hatte ich ja auch schon. In Konstanz – und das war auch der Grund für das spezielle Programm – wurde eine ganz bestimmte Richtung von Wissenschaftsphilosophie vertreten, nämlich die konstruktivistische Wissenschaftstheorie.

**MG**: Vielleicht direkt anschließend nochmal: Jetzt sind bei Dir Biologie, Philosophie und auch Anthropologie auf dem Tisch, aber die Wissenschaftsgeschichte als Disziplin ist noch nicht vorgekommen. War sie Dir überhaupt ein Begriff? Dass es vereinzelte Lehrstühle gab, in Deutschland oder Italien?

**SdC**: Ja, aber was ich davon gesehen hatte, schien nicht sehr interessant zu sein. Es war noch keine theoretisch fundierte Wissenschaftsgeschichte, sondern mehr in dem Stil „Wer hat was wann gemacht“. Das wurde uns auch in den Vorlesungen so vorgeführt. Aber ich glaube, ich habe einfach noch nicht gesehen, wie man das interessant gestalten konnte. Wenn man über Wissenschaft nachdenken wollte, dann war Philosophie zu der Zeit in Deutschland die Disziplin dafür. Allerdings habe ich nach dem Biologiestudium auch an einen Studiengang über *science policy* in Sussex gedacht, aber das war nicht praktikabel.

**AtH**: Um die Frage von Mathias noch weiterzutreiben: Welche Wissenschaftsgeschichte war dann zum ersten Mal interessant für dich, so dass Du dachtest, okay, ich kann die Biologie und die Philosophie mitnehmen und mache aber trotzdem was Neues, nämlich Wissenschaftsgeschichte? Wann und wo, würdest Du sagen, begann es, dass dieses „Daten, Erfinder und große Forscher“ nicht mehr im Vordergrund stand, sondern eine Wissenschaftsgeschichte, von der Du dachtest: Die ist wirklich interessant!

**SdC**: Na ja, mein Philosophiestudium und die sehr lange Auseinandersetzung mit der Philosophie von Maurice Merleau‐Ponty spielten da eine wichtige Rolle. Aber wenn wir mal darüber hinweggehen, dann fällt mir gegen Ende dieser Zeit in Konstanz unsere kleine Lesegruppe ein. Eine kleine Lesegruppe, bestehend aus Lorraine Daston,^10^ die damals zwischen Konstanz und Brandeis pendelte, Catherine Wilson,^11^ einer Fulbright‐Stipendiatin aus Amerika, einer weiteren PhD‐Mitstreiterin in der Philosophie, Christine Schildknecht,^12^ und mir. Wilson hatte uns zusammengebracht. In dieser Gruppe haben wir Wissenschaftsgeschichte gelesen.

Es war wirklich Daston, die mich davon überzeugt hat, dass Wissenschaftsgeschichte mein Fach ist, weil ich da alle meine Erfahrungen – von der Biologie bis zur Philosophie –, ebenso meine Sprachkenntnisse zusammenbringen könnte. An dem Punkt hatte sich Wissenschaftsgeschichte eben auch verändert und das Walther‐Rathenau‐Programm war ein seit 1989 bestehendes Programm, um diese neuen Ansätze der Wissenschaftsgeschichte nach Deutschland zu bringen. Ich habe von Daston erfahren, dass diese Fellowships das erste Mal ausgeschrieben werden und mich beworben. Das Programm richtete sich übrigens explizit an Bewerber:innen, die aus anderen Disziplinen kamen und noch keine wissenschafts‐ oder technikgeschichtliche Spezialausbildung hatten. So kam ich in die Wissenschaftsgeschichte. Ich war zu diesem Zeitpunkt schon ziemlich sicher, dass ich nicht mehr ins Labor zurück, aber auch nicht in der Philosophie weitermachen wollte. Aber diese Wissenschaftsgeschichte, die schien mir attraktiv. Es war ja auch erstmal ein Jahr, das konnte man ja mal ausprobieren …

Aber auch in dieser Phase, also *post‐PhD*, hatte ich wieder alle möglichen Ideen, was ich sonst noch so machen könnte. Zum Beispiel habe ich damals ziemlich stark mit dem Öko‐Institut zusammengearbeitet und mich dort auch auf eine neue Stelle zum Thema Gentechnologie beworben. Es ging immer darum, wie man Wissenschaft kritisch betrachten and anwenden konnte. Das Öko‐Institut war eine gute und wichtige Erfahrung, wo ich an vielen Diskussionen teilgenommen und an mehreren Projekten mitgearbeitet habe. Und es gab damals die Idee der Wissenschaftsläden, also der Einsatz von Wissenschaft für gesellschaftliche Auseinandersetzungen. Das schien mir alles sehr spannend zu sein, Wissenschaft zu benutzen, aber nicht sie unbedingt selbst zu machen, also nicht im Labor zu sein und da immer weiter zu basteln.

**AtH**: Es ging also darum, Wissenschaft im emanzipativen Sinne zu verstehen – Stichwort Wissenschaftsläden oder das Öko‐Institut, das in Konstanz war, richtig?

**SdC**: Es war in Freiburg, auch wenn ich damals schon nach Konstanz gewechselt hatte. Das Öko‐Institut hatte seinen Anfang darin, Bürgern, die gegen die Errichtung von Kernkraftwerken protestierten, die wissenschaftliche Expertise zu liefern, die sie für ihren Widerstand und vor Gericht benötigten. Später hat sich dann das Themenspektrum geweitet: Chemie kam zuerst hinzu, und da habe ich am Projekt *Chemie im Haushalt* mitgearbeitet,^13^ dann die Gentechnik. Die Mitarbeit und die Diskussionen im Öko‐Institut in dieser Zeit, das heißt während ich in Philosophie promovierte, waren ganz wichtig für mich – gerade auch wegen der Überlegungen, wie kritische Wissenschaft zu betreiben ist. Sehr engagierte Leute, sehr, ja …

**AtH**: … einfach auch eine sehr politische Position in diesem Studium und über dieses Studium hinaus. Die Frage, die ich eigentlich vorhin damit verknüpfen wollte, war, ob Du Dich daran erinnerst, was ihr in dieser Gruppe mit Daston, Wilson und Schildknecht gelesen habt? Ob da zum Beispiel für Euch auch schon so etwas auftauchte wie Feministische Wissenschaftstheorie, oder ob Ihr gesagt habt, wir lesen jetzt Grundlagentexte? Wie habt Ihr das aufgezogen?

**SdC**: Ja, das hätte ich jetzt auch gerne genauer gewusst [lacht]. Also, genau weiß ich es nicht mehr, aber vorwiegend haben wir unsere eigenen Texte gelesen, an denen wir gerade arbeiteten. Es ging um mikroskopische Beobachtung – das war Cathys Thema –, über Experimente und die Geschichte von *facts* – daran hat Raine gearbeitet, auch über das Sammeln. Christiane und ich unterrichteten damals ein Seminar über Bacon. Das waren also durchaus Themen, die sich mit der neuen Historiographie auseinandersetzten, aber vielleicht eher unter der Rubrik Historische Epistemologie zu fassen wären. Für mich war das alles neu und spannend. Übrigens hatte die Gruppe auch eine soziale Funktion. Drei von uns hatten damals kleine Kinder, was in der *academia* für Frauen noch nicht so üblich war.

**MG**: Wir sind zweimal schon über Merleau‐Ponty hinweggesprungen, einmal nach vorne und einmal nach hinten. Jetzt würde mich aber doch interessieren, wie er in Deine Entwicklung hineinpasst, und welche Funktion das für Dich im Nachhinein hatte.

**SdC**: Also, ich habe über den französischen Phänomenologen promoviert und praktisch an Merleau‐Ponty mein ganzes Philosophie‐Studium aufgehängt. Denn um Merleau‐Ponty zu verstehen, musste man viel Philosophiegeschichte verstehen. Ich weiß nicht mehr ganz genau, wie ich auf ihn kam, es hatte nichts damit zu tun, was in Konstanz lief. Da wurde mir immerfort die Frage gestellt: „Warum Merleau‐Ponty?“ Dort wurde auf der einen Seite konstruktivistische Wissenschaftstheorie betrieben, also ein Nachdenken über Wissenschaft, aber in einer anderen Tradition. Auf der anderen Seite war die Frankfurter Schule vertreten, mit Albrecht Wellmer,^14^ der mein Haupt‐Doktorvater war. Mein Zweitbetreuer war Jürgen Mittelstraß.^15^ Aber in der Frankfurter Schule, so schien mir, wurde hauptsächlich über Sozialwissenschaften nachgedacht, und die Naturwissenschaften wurden eher als Folie dagegengehalten, weil sie eben anders funktionierten. Ich war ziemlich naiv als ich damit begann, aber Merleau‐Ponty hat mich interessiert, weil er sich als Philosoph aktiv mit den Naturwissenschaften seiner Zeit auseinandergesetzt hat. Er spielt immer wieder das naturwissenschaftliche Denken gegen das transzendentale Vorgehen und Nachdenken über Wissenschaft aus, und es geht auch um Descartes’ Dualismus von Körper und Geist, der die Spaltung von Wissenschaft und Philosophie untermauert. An dieser Stelle setzt Merleau‐Ponty die Idee des Leibes (oder des lebendigen Körpers), mit dem wir in der Welt sind, die auch seiner Wahrnehmungstheorie zugrunde liegt. Das hat mich alles fasziniert. Letztlich habe ich Merleau‐Ponty dann so interpretiert, dass er eine Art Wende zur Sprache und Geschichte eingeläutet hat.

So bin ich auch zur Überzeugung gekommen, dass man in die Geschichte schauen muss, um zu verstehen, was Wissenschaft ist und wie die Wissenschaft über die Welt redet, dass man das geschichtlich anpacken muss. So beschreibt auch das letzte Kapitel meiner Dissertation, die schließlich als Buch erschien,^16^ die Berührungspunkte zwischen Merlau‐Pontys Theorie der Geschichte und der Wahrheit und den Arbeiten von Foucault, Kuhn und anderen Wissenschaftstheoretikern, besonders in der französischen epistemologischen Tradition. Aber Merleau‐Ponty ist nur ein Teil der Geschichte. Hinzu kam für mich die Wende zur Praxis in der Wissenschaftsgeschichte. Das zusammen machte für mich wirklich sehr viel Sinn. Die Praxis‐Wende war ein dominantes Thema, als ich begann, mich für Wissenschaftsgeschichte zu interessieren, eben hauptsächlich in Berlin zur Zeit der Wende (1989–1991). Dazu wollt Ihr vermutlich mehr hören, oder?

**AtH**: Genau, hier kommt nämlich der Verbund für Wissenschaftsgeschichte und das bereits genannte Walther‐Rathenau‐Programm ins Spiel. Erzähle uns doch, wie Du persönlich dazu gekommen bist – Daston hat Dich scheinbar darauf aufmerksam gemacht, wenn ich Dich vorhin richtig verstanden habe –, aber auch, wie Du den Anfang, die Genese dieses Verbundes wahrgenommen hast. Denn der stand ja in direktem Zusammenhang mit dem Wissenschaftskolleg (Wiko).

**SdC**: Ich glaube, dass dieser Verbund und das ganze Rathenau‐Programm eine sehr interessante Initiative war, die vieles zusammenbrachte. Es war ja der Versuch, die neuen Ansätze der Wissenschaftsgeschichte nach Deutschland zu bringen. Dafür wurden die Rathenau‐Fellowships eingerichtet, die von der Volkswagen‐Stiftung finanziert waren. Es sollten fünf Fellows pro Jahr sein, aber im ersten Jahr waren wir nur vier. Die anfängliche Finanzierung lief für fünf Jahre. Diese Fellowships wurden vom Verbund für Wissenschaftsgeschichte und einem internationalen Netzwerk getragen. Der Verbund für Wissenschaftsgeschichte wiederum wurde vom West‐Berliner Senat unterstützt und brachte viele wissenschafts‐ und technikgeschichtliche Institutionen in West‐Berlin zusammen. Dazu gehörten Institute der TU und FU, das Museum für Verkehr und Technik, das Archiv der Max‐Planck‐Gesellschaft, die Historische Kommission, das Wissenschaftszentrum Berlin für Sozialforschung (WZB), in dem wir uns oft getroffen haben, und das Wissenschaftskolleg im Grunewald. Wie du schon andeutetest, Anke, Wolf Lepenies,^17^ der damals Direktor des Wiko und auch sonst wissenschaftspolitisch sehr aktiv war, war zusammen mit anderen maßgeblich an der Organisation des Ganzen beteiligt. Zusätzlich zum Verbund gab es dann noch ein internationales Netzwerk, dem neben Lepenies Tim Lenoir,^18^ der damals in Stanford lehrte und Berlin oft besuchte, Daston aus Brandeis, Simon Schaffer aus Cambridge,^19^ Tom Hughes aus Philadelphia,^20^ und Yehuda Elkana^21^ aus Tel Aviv angehörten. Das waren auch die Partneruniversitäten, und es gehörte zum Programm, dass wir Fellows eine gewisse Zeit in einer oder mehrerer dieser Partneruniversitäten verbringen würden, was ich intensiv genutzt habe. Dieses Programm kann auch als ein Versuch verstanden werden, die Ausbildung von Postdoktorand:innen oder Nachwuchswissenschaftler:innen in Deutschland neu zu gestalten, und zwar speziell für die Geisteswissenschaften, wo solche Ansätze noch ganz fehlten.

Das Programm war interdisziplinär, interinstitutionell und international aufgestellt, auf vielen Ebenen wurde da versucht, etwas Neues mit neuen Inhalten zu machen. Wissenschaftsgeschichte wurde weit gefasst, als ein Fach, das sach‐ und gesellschaftliches Wissen verband und einen interdisziplinären Zugang geradezu erforderte. Uns wurde damals beim *welcome* im Oktober 1989 vermittelt, dass wir Teil dieser *exciting* neuen Initiative waren, von der man sich viel versprach.

Der Verbund und die Koordinationsstelle für das Stipendienprogramm waren an der TU angesiedelt, wo es ein Sekretariat gab. Im ersten Jahr betreute Christoph Meinel^22^ als Sekretär – heute würde man vielleicht eher Koordinator sagen – das Programm, später übernahm Michael Becker^23^ diese Aufgabe. Wir trafen uns regelmäßig für Seminare und Workshops, oft mit auswärtigen Gastprofessor:innen. Ich erinnere mich an Workshops mit Simon Schaffer, Norton Wise, Tom Hughes, Herbert Mehrtens^24^ und vielen anderen. Außerdem war jeder von uns Fellows einem Verbund‐Institut zugeordnet, das inhaltlich am besten zu unseren jeweiligen Projekten passte. Mit meinem Projekt in der Geschichte der Botanik wurde ich dem Institut für Medizingeschichte zugeordnet, das damals noch unter Rolf Winau^25^ in Steglitz arbeitete. Ich wurde sehr freundlich aufgenommen und habe viel gelernt. Neben Winau waren Heinz‐Peter Schmiedebach^26^ und Michael Hubenstorf,^27^ die dort habilitierten, wichtige Gesprächspartner. Auch Volker Hess,^28^ der an seiner Doktorarbeit schrieb, lernte ich so kennen. Also, das war eine wichtige institutionelle Verbindung. Auch ganz wichtig waren die Sommerakademien. Anke, Du warst doch auch mal Fellow?

**AtH**: Ja, ich bin 1994 Rathenau‐Fellow geworden und habe ab 1992 an den Sommerakademien teilgenommen. Für mich war es der Einstieg in die Wissenschaftsgeschichte, ich wusste vorher gar nicht, was das ist. Ich hatte zwar das Medizinhistorische Institut in Lübeck kennengelernt und dort als Hilfskraft für Hans‐Jörg Rheinberger^29^ gearbeitet, aber keine richtige Vorstellung vom internationalen Zusammenhang, und diese Sommerakademien waren total wichtig.

**SdC**: Die erste Sommerakademie, die ich mitgemacht habe, war 1990 zum Thema „Die Produktion der Moderne“. Noch wichtiger für mich war dann die Akademie im folgenden Sommer über „Schreiben und Beschreiben in der Wissenschaft“. Warst Du da schon dabei?

**AtH**: Nein, ich glaube, damals war ich zu einem einzelnen Vortrag da, oder irgendwie so etwas. Ich erinnere mich dunkel daran, aber habe es noch nicht wirklich verstanden.

**SdC**: Diese Sommerakademien waren drei Wochen lang …

**AtH**: Sie waren *irre* lang, aber zumindest zu meiner Zeit dauerten sie zwei Wochen.

**SdC**: Ja, mit Top‐Leuten und eben dieser Gruppe von jungen Nachwuchswissenschaftlern, ich glaube Doktorand:innen und Postdocs … Sie waren ziemlich offen, weil es darum ging, diese neuen Ansätze zu verbreiten.

**MG**: Zwei Fragen von einem Nachgeborenen: Zum einen, waren die sogenannten Rathenau‐Fellows im Bereich Wissenschaftsgeschichte zugleich die Fellows des Verbundes?

**SdC**: Ja, genau. Dieses Fellowship‐Programm lief erstmal für fünf Jahre, danach wurden die Rathenau‐Fellows in das 1994 neu gegründete Max‐Planck‐Institut für Wissenschaftsgeschichte (MPIWG) eingebunden. War das so in Deinem Fall, Anke?

**AtH**: Ich war schon halbe‐halbe, der Übergangsjahrgang 1994/95, der sowohl im Verbund als aber auch am Max‐Planck‐Institut (MPI) beheimatet war.

**SdC**: Ich weiß nicht, ob es diese Fellowships jetzt noch gibt.

**MG**: Die andere Frage: Wie passte die Entstehung dieses extrem heterogenen und in seiner Anlage sehr ambitionierten Verbundes für Wissenschaftsgeschichte in die Wissenschaftspolitik West‐Berlins in den späten 80er‐Jahren? Hat es einen Grund, dass er damals und dort entstand, hing das etwa an einzelnen Leuten oder lässt sich das irgendwo anders festmachen?

**SdC**: Lepenies, der ja schon früh über Wissenschaft in Zusammenhang mit Gesellschaft, Kultur und Politik nachgedacht und publiziert hat, und das von ihm geleitete Wiko waren, glaube ich, entscheidend. Verschiedene der internationalen Wissenschaftler:innen, die zentral an dieser Initiative beteiligt waren, waren auch schon in Berlin gewesen, unter anderem am Wiko. Dazu gehörten Lenoir, Hughes und Elkana, der ja später – oder vielleicht schon damals – ein *permanent fellow* am Wiko war. Man müsste das nochmal genauer nachprüfen, aber es ist wahrscheinlich, dass der Plan damals entstand und eben deshalb auch in Berlin realisiert wurde, wo es eine Vielzahl relevanter Einrichtungen und wegen der reichen institutionellen Geschichte auch viele Quellen gab. Hughes und Lenoir arbeiteten auch selber über Aspekte der deutschen Wissenschafts‐ und Technikgeschichte. Die Bemühungen, den Verbund aufzustellen, begannen, meine ich, 1987/88 und wir fingen 1989 im Herbst an, aber in dem Sommer gab es schon ein Kolloquium über Rathenau und die Moderne oder so ähnlich, mit dem der Verbund sich vorstellte. Dieses Kolloquium und dann auch der festliche Empfang für die ersten Stipendiat:innen im Herbst fanden am Wiko statt, die Seminare oft im WZB, und das Sekretariat war an der TU. So hat das Programm wirklich viele Institutionen zusammengebracht.

**MG**: Tauchte Nicolaus Sombart^30^ vom Wiko in dem Zusammenhang auf?

**SdC**: Lepenies war für mich die wichtige Person dort.

**AtH**: Und Hughes war auch nicht unwichtig, sonst wäre das Museum für …

**SdC**: Sehr wichtig!

**AtH**: … Verkehr und Technik nicht Teil des Verbundes gewesen und – deshalb auch dieses interdisziplinäre Programm – die Figur Walther Rathenau, die für alle gleichermaßen Bedeutung hatte, so etwas wie eine initiale Figur.

**SdC**: Ich glaube Rathenau war eine *besonders* wichtige Person für Hughes, der Technikwissenschaft gemacht hat, und eben auch eine Schlüsselfigur für das Programm, wie Du sagst, Anke, weil er Ingenieur, Industrieller und Politiker, alles in einem war, oder eben ein Bildner von wissenschaftlich‐technischen und gesellschaftlichen Systemen, wie sie für die Moderne charakteristisch waren. Um ihn handelte ja auch das erste Kolloquium des Verbundes und dann auch noch teilweise die erste Sommerakademie.

Lepenies und Hughes, vielleicht noch Lenoir, der damals häufig Berlin besuchte, waren, soweit ich weiß, die tragenden Leute des Programms, organisatorisch wie inhaltlich, und um in Deutschland eine neue Wissenschaftsgeschichte zu verankern. Dazu kamen dann noch Schaffer und Daston, aber ich hatte nicht das Gefühl, dass sie so zentral involviert waren, auch wenn sie für mich wichtige Personen waren oder dann wurden.

**AtH**: So erinnere ich es auch.

**SdC**: Von unserer peripheren Perspektive als Fellows sah es dann so aus, dass sobald die Mauer gefallen war – und das passierte ein paar Wochen nach der Einweihungsfeier – dieselben Personen sehr bald weitergedacht haben, wie die neuen politischen Möglichkeiten der Öffnung nach Osten wissenschaftspolitisch genutzt werden konnten. Alle Anstrengungen schienen irgendwie in diese Richtung zu gehen. Kleine Anfänge von dieser Vereinigung haben wir auch mitgekriegt. Wir fingen an, uns manchmal gemeinsam mit ostdeutschen Kolleg:innen zu treffen. Vielleicht weißt Du das, Anke, es war ein Raum jenseits des vom Westen wieder zugänglichen Gendarmenmarktes.

**AtH**: Ja, in der Jägerstraße. Dort war der Forschungsschwerpunkt Wissenschaftsgeschichte der Akademie der Wissenschaften der DDR angesiedelt.

**MG**: Bestanden denn sofort Kontakte zu den Netzwerken in West‐Berlin, über die wir in anderen Interviews bereits gesprochen haben, etwa mit Rheinberger oder Peter McLaughlin,^31^ wie auch Wolfgang Lefèvre,^32^ und dem dort erwähnten Seminar für Begriffsgeschichte an der FU Berlin oder war das wie Ost‐Berlin auch zunächst eine fremde Welt?

**SdC**: Das Seminar habe ich besucht, und zwar wegen eines Zufalls. Wolfgang Lefèvre und Ursula Klein^33^ waren beide in Konstanz in dem Jahr, als ich auch noch dort war. Als es dann akut wurde mit meiner Fellowship‐Bewerbung, erinnere ich mich noch, wie Wolfgang sagte: ‚Ja, wenn Du nach Berlin gehst, dann musst Du in dieses Seminar gehen.“ Das war tatsächlich ein Ort in Berlin, wo schon seit den späten 1960er oder 1970er Jahren über Wissenschaftsgeschichte nachgedacht wurde, nicht wahr? Aber ich habe von Anfang an das Gefühl gehabt, dass da vielleicht, *maybe*, sogar ein bisschen eine Animosität bestand, denn ich denke nicht, dass es anfänglich Verbindungen gab zu dem Verbund. Ich meine mich zu erinnern, dass ich der einzige Fellow war, der eben wegen dieser persönlichen Kontakte daran teilgenommen hat. Die Ansätze waren sehr anders, aber ich habe da interessante Leute kennengelernt, unter anderem auch Jürgen Renn,^34^ der später einer der Direktoren des neugegründeten MPI für Wissenschaftsgeschichte geworden ist und auch mehrere aus dem Seminar ans Institut geholt hat. Ich glaube, dass der Verbund ein wichtiges Sprungbett für diesen neuen Plan des MPIWG war, auch wenn ich zu dem Zeitpunkt nicht mehr in Berlin lebte. Zur Illustration eine Erinnerung zur Berliner Situation in den frühen 1990ern: Ich habe in einem „Ost‐West“‐*folder,* in meinem *filing cabinet* [lacht], einen Artikel von Lepenies mit dem Titel „Wissenschaftspolitik der Stärke“^35^ gefunden, und der sprach eben davon, dass man das Zusammenwachsen der beiden Stadthälften als Chance sehen sollte für eine neue Wissenschaftspolitik. Dabei nannte er *explizit* das Beispiel Wissenschaftsgeschichte und redete dann von dem Verbund als einem Beispiel eben einer solchen Wissenschaftspolitik. Wenige Jahre später gab es das Max‐Planck‐Institut, das diese Ideen zumindest teilweise verwirklichte. Das fand ich interessant. Es spielte sicher eine Rolle – ob es die entscheidende Rolle für die Gründung des MPIWG gespielt hat, weiß ich nicht.^36^

**MG**: Was war denn für Dich der wichtigste Impuls oder persönliche Gewinn aus dieser Berliner Zeit?

**SdC**: Mich hat das Jahr des Fellowships für die Wissenschaftsgeschichte eingenommen. Ich habe am Ende des Jahres eine Amerika‐Reise angehängt, wo ich an der University of Pennsylvania bei Hughes war, und auch mehrere Wochen in Stanford, wo Lenoir, aber auch Peter Galison,^37^ Nancy Cartwright^38^ und andere tätig waren. Am Ende habe ich noch an meinem ersten History of Science Society‐Meeting in Seattle teilgenommen. Das hatte mir Robert Kohler,^39^ den ich in Philadelphia kennenlernte, nahegelegt. Für mich war klar, das war spannend, aber ich brauchte mehr Zeit und bin dann einfach noch ein zweites Jahr geblieben und war Teil des Programms, ohne dass ich offiziell ein Fellow war. Für mich entstand das Gefühl, dass ich hier meine Interessen zusammenbringen konnte.

Aber etwas habe ich noch nicht erwähnt. Einer meiner Co‐Fellows war Andy Warwick.^40^ Warwick war, anders als wir restlichen drei, wissenschaftshistorisch ausgewiesen. Er hatte in Cambridge bei Schaffer promoviert. Er hat mich mit Literatur gefüttert und dabei immer gesagt: „You have to read *this*, you have to read *this*, and *this* …” Da waren Artikel und Bücher von Schaffer dabei, von Steve Shapin, Harry Collins, Bruno Latour, all das. Das haben wir teilweise auch in den Seminaren diskutiert. Schaffer zum Beispiel war einer von den Leuten, die zu uns kamen und Workshops gemacht haben. Aber die intensiven Diskussionen mit Andy über das ganze Jahr hinweg waren wichtig, zusätzlich zu allen Seminaren, Vorträgen und so weiter. Das fand ich auf jeden Fall spannend und es war auch ein Moment, an dem die Wissenschaftsgeschichte im Umbruch stand und es sehr viele Grundlagendiskussionen gab, über die Wende zur Praxis zu Fragen: Was ist Wissenschaft? Was Experiment? Theorie? Wie entstehen Tatsachen? Das passte doch sehr gut für jemanden, der aus der Philosophie kam. Das waren Fragestellungen zu denen ich Bezugspunkte hatte. Eine der ersten größeren internationalen Konferenzen, an denen ich teilnahm, war über *tacit knowledge* und *skills* in der experimentellen Praxis in Bath. Da wurde sogar Merleau‐Ponty zitiert.

**AtH**: Das heißt, Du bist dann ein zweites Jahr in Berlin geblieben, hast Dich finanziell irgendwie über Wasser gehalten und in der Zeit Dein Rathenau‐Fellow‐Projekt vorangetrieben und Veröffentlichungen über Botanik im 19. Jahrhundert herausgebracht? Stand das dann im Vordergrund oder ging es eher darum, Berlin als einen Ort zu sehen, wo Du für die Wissenschaftsgeschichte oder wo Du über die Wissenschaftsgeschichte noch etwas lernen, erfahren konntest?

**SdC**: Ich würde sagen beides. Zum Beispiel habe ich auch meine Dissertation überarbeitet, die 1990 als Buch herauskam. Ich war irgendwie immer sehr beschäftigt. Und dann kam ja die Sommerakademie. Das Interesse für die Botanik und Pflanzenphysiologie führte sehr schnell zu einem Interesse für Kymographen und selbstschreibende Geräte. Darüber habe ich dann in der zweiten Sommerakademie vorgetragen, die über das Thema „Schreiben und Beschreiben in der Wissenschaft“ ging. Die Bibliothek am Botanischen Institut war sehr wichtig, da habe ich sehr viel Archivmaterial gefunden, und dann war ich auch im Sachs‐Archiv in Würzburg und in Cambridge, eine der Partnerinstitutionen, um an den Darwin‐Briefen zu arbeiten. Das war mein erster Besuch in Cambridge.

Wie ich das finanziell gemacht habe, ist eine interessante Frage. Ich habe eigentlich immer gearbeitet, hatte kein Stipendium mehr. Irgendwie hatte ich mir Geld gespart. Es war dann ja auch nicht mehr so lange, eigentlich nur ein halbes Jahr, weil ich den Herbst in Amerika verbracht hatte und dann war es praktisch eigentlich nur von Dezember bis Juni. Dann bekam ich ja die Postdoc‐Stelle an der Wellcome Unit in Cambridge.

**MG**: Und dann kamst Du wieder zur Molekularbiologie.

**SdC**: Ja. Davor ging es, wie gesagt, um Pfanzenphysiologie, Sachs, Darwin, die Art des richtigen Experimentierens, darum ging es in dieser Kontroverse, und dazu das Interesse für selbstschreibende Geräte … Ich fand das interessant, aber man hat mir nahegelegt, dass, wenn ich weiter in der Wissenschaftsgeschichte bleiben wollte, was nicht unbedingt Ziel dieses Rathenau‐Fellowships war … Die Erwartung war ja eher, dass man zurück in seine ursprüngliche Disziplin gehen und dort die neuen Fragestellungen über Wissenschaft und Technik einbringen sollte, was eine interessante Idee ist, nicht? Kurzum, man legte mir also nahe: Wenn ich weiter Wissenschaftsgeschichte betreiben wollte, dann sollte ich doch mein molekularbiologisches Wissen nutzen und in dieser Richtung forschen. Aber daraus wäre nichts geworden, wenn nicht zufällig an der Wellcome Unit in Cambridge eine Projektstelle für die Geschichte der Molekularbiologie in Cambridge ausgeschrieben worden wäre, und die habe ich bekommen. Das war ein glücklicher Umstand, weil ich zu der Zeit noch nicht sehr stark als Wissenschaftshistorikerin ausgewiesen war. Aber dort habe ich angefangen, mich mit der Wissenschaftsgeschichte der Molekularbiologie zu beschäftigen. Die Publikationen über Sachs und Darwin kamen teilweise erst später, 1992 bzw. 1994, heraus, nicht sofort.

Diese Berliner Jahre … Es gab also das Seminar für Begriffsgeschichte, es gab die Verbindung zur Medizingeschichte, hinzu kam alles, was im Verbund lief – was sehr viel war – aber auch zum Beispiel Rheinberger. Ihn habe ich kennengelernt, weil Lenoir mir gleich am Anfang sagte: „You have to get to know Hans‐Jörg.“ Der war zu jener Zeit in Stanford, wo er *something very cool* machte, so Lenoir, nämlich eine Derrida'sche Analyse der Experimentalforschung am Beispiel der Molekularbiologie.

**AtH**: Experiment, Differenz, Schrift.^41^

**SdC**: Ja, daran arbeitete er. Hans‐Jörg kam manchmal nach Berlin und so habe ich ihn dann getroffen. Er schickte mir einen *early draft* von seiner Arbeit, den wir diskutierten. So kam der Kontakt mit Hans‐Jörg zustande, ganz klar über den Verbund und das Kolleg. Es war sehr ungewöhnlich, mit wie vielen auch *senior international scholars* wir damals in Kontakt kamen. Das war sehr anders als während meines Philosophie‐Studiums. Bevor man promoviert war, ging man damals sowieso nicht zu Meetings. Erstmal musste man was vorlegen. Das Rathenau‐Programm war in der Hinsicht wirklich revolutionär. Auch unter den Fellows habe ich Leute kennengelernt, mit denen ich nachher noch lange in Verbindung stand. Im zweiten Jahr, wer war denn dann … ich glaube, da war auch Skúli Sigurdsson^42^ Fellow, vielleicht sogar mit Dir, Anke?

**AtH**: Nein, er war vor mir Fellow, ich glaube auch Sybilla Nikolow^43^ war vor mir.

**SdC**: Ja, dann auch Bob Brain,^44^ Bill Clark,^45^ das sind alles Leute, die ich im zweiten, oder vielleicht im dritten Jahr kennenlernte. Ich erinnere mich nicht, ob ich dann weiter zu den Sommerakademien gegangen bin.

**AtH**: Ich erinnere mich an Dich! Weil ich Dich auf einer Sommerakademie kennengelernt habe. Und in Cambridge.

**SdC**: Und nicht in Lübeck?

**AtH**: Ahhh, Du hast Recht. Ich habe Dich in Lübeck kennengelernt. Ja sicher!

**SdC**: Denn die Lübecker Meetings waren ja auch sehr wichtig. Wann war das erste?

**AtH**: Die erste Tagung von Michael Hagner,^46^ Hans‐Jörg Rheinberger und Bettina Wahrig‐Schmidt^47^ fand 1990 zu Johannes Müller statt, daraus wurde der Johannes‐Müller‐Band.^48^ Da kannte ich die anderen Lübecker aber noch nicht. Ich bin nur zu dieser Tagung hingegangen, weil ich gerade frisch in Lübeck angekommen war, im Krankenhaus gearbeitet habe, aber das Thema so interessant war. Und 1991 fand die Tagung statt, bei der wir uns richtig kennengelernt haben, zum Thema „Experimentalisierung des Lebens“.

**SdC**: Ja, da habe ich dann vorgetragen über die graphischen Systeme.

Und dann gab's noch was anderes, was wichtig war: eine Sommerschule in Uppsala. Ich glaube das war auch 1990 im Sommer oder 1991. Die hat Meinel mir nahegelegt, also muss es 1990 gewesen sein. Diese Internationale Sommerschule für Wissenschaftsgeschichte hatten Tore Frängsmyr^49^ von Uppsala, John Heilbron^50^ von Berkeley und Giuliano Pancaldi^51^ aus Bologna ins Leben gerufen. Später kam Dominique Pestre^52^ dazu. Die Schule fand jedes zweite Jahr in einer der Partneruniversitäten statt, 1990 eben in Uppsala. In dem Jahr ging es um die Geschichte der Biologie. Das war in einer Zeit, als die Geschichte der Physik noch sehr stark dominierte. Larry Holmes^53^ gehörte auch zu den Vortragenden.

Diese Sommerschule bedeutete eine ganze Woche intensive Auseinandersetzung mit Wissenschaftsgeschichte und ihren Methoden. Und dann gab es auch ein wichtiges Meeting in Trento, organisiert von Renato Mazzolini,^54^ zu *non‐verbal communication in science.* Da habe ich ebenfalls über die selbstschreibenden Geräte in der Pflanzenphysiologie vorgetragen. So kamen schließlich auch die ersten wissenschaftsgeschichtlichen Publikationen zustande.^55^

**MG**: Zurück nach Cambridge. Dort hattest Du auch einen klaren Auftrag: Du warst vom Wellcome‐Trust angestellt, um Geschichte der Molekularbiologie zu betreiben. Wie hast Du geschafft, das, was Dich umgetrieben hat und Du selber machen wolltest, mit der – ich will nicht sagen – Auftragsgeschichtsschreibung zusammenzubringen?

**SdC**: Ich würde wirklich nicht Auftragsgeschichtsschreibung sagen. Es gab eine Wellcome Unit für History of Medicine, die dem Department for History and Philosophy of Science (HPS) angegliedert war, aber sie war *separately* *funded*. Die Unit hat die Initiative ergriffen und für zunächst drei Jahre eine Forschungsstelle ausgeschrieben. Diese Stelle habe ich bekommen. Zu der Zeit war das Projekt noch vollkommen unabhängig vom Laboratory of Molecular Biology (LMB). Und wie ich die Geschichte der Molekularbiologie in Cambridge anpacken würde, darin war ich total frei, da hat mir niemand reingeredet. Aber ich habe ziemlich früh den Plan entwickelt, dass ich diese neuen Ansätze, von denen ich einiges aufgenommen hatte, dass ich die auf die Geschichte der Molekularbiologie anwenden würde. Also, dass ich sozusagen die Molekularbiologie für diese Ansätze öffnen würde.

Damals gab es Institutions‐ und Disziplinengeschichte, die noch sehr stark vertreten waren, und dann eben die neueren *laboratory studies*, wo es um wissenschaftliche Praxis ging. Man könnte ja meinen, diese alte Disziplinen‐ und Institutionsgeschichte brauchten wir jetzt nicht mehr, jetzt schauen wir ins Labor und untersuchen, was die Leute da wirklich machen und wie das funktioniert. Aber mir schien, dass man diese beiden Ansätze irgendwie zusammenbringen sollte. Mein Buch ist der Versuch, das zu tun, also das Machen einer bestimmten Wissenschaft in einer gewissen Institution in einem bestimmten historischen Moment anzuschauen, aber auch gleichzeitig sehr genau zu beobachten, was in diesen Laboren passiert.^56^ Also diese Sachen zusammenzubringen. Diese Vision hatte ich, glaube ich, schon recht früh. Ich meine, es gab ja schon erste *accounts* der Geschichte der Molekularbiologie. Die meistzitierten Werke damals waren *The Eighth Day of Creation: Makers of the Revolution in Biology* (1979) von Horace Judson, einem Wissenschaftsjournalisten, der die Pioniere der Wissenschaften interviewt hatte, und Robert Olbys *The Path to the Double Helix: The Discovery of DNA* (1974). Gerade über James Watson und Francis Crick und ihre berühmte Zusammenarbeit, die ja in Cambridge stattfand, war also schon einiges geschrieben worden. Ich wollte erstmal weiterschauen und mir andere Fragen vornehmen.

Zu meiner Postdoc‐Stelle gab es auch ein Advisory Comittee, zu dem gehörte John Kendrew,^57^ der im selben Labor wie Crick und Watson an der Struktur von Proteinen gearbeitet hatte und auch im selben Jahr, zusammen mit Max Perutz, den Nobelpreis bekam. Es ist übrigens nicht ausgeschlossen, dass die Idee des Projekts einer Geschichte der Molekularbiologie in Cambridge von ihm stammte, weil er historisch sehr interessiert war (er war nach seiner Emeritierung wieder zurück nach Cambridge gezogen). Bei einem Empfang einige Monaten nachdem ich meine Projektstelle begonnen hatte, traf ich Kendrew, und er fragte, ob ich denn mit meiner Arbeit angefangen hätte und warum ich noch nicht mit ihm gesprochen hätte. Es war, als ob er erwartete, dass ich mit ihm über die Geschichte spreche, und natürlich hatte er auch bereits sein gesamtes umfangreiches Archiv der Bodleian Library in Oxford hinterlassen. Damit habe ich dann angefangen. Er verschuf mir Zugang zum 900‐seitigen Katalog seiner Papiere, der außerordentlich hilfreiche Einführungsparagraphen zu jedem Abschnitt hatte, die die Archivarin Jeannine Alton, ich vermute in enger Zusammenarbeit mit Kendrew, verfasst hatte. Für mich war Kendrew eine wichtige Figur, weil seine Karriere stark von seiner Erfahrung als Wissenschaftler im Krieg geprägt war. Er hat mich wirklich auf dieses Thema gebracht. Er hatte als junger *scientist* diese *war experience* durchgemacht, ging dann zurück ins Labor, hat in der Proteinkristallographie gearbeitet und dann relativ bald die experimentelle Wissenschaft aufgegeben und sich der Wissenschaftspolitik zugewendet. Er hat sich sehr eingesetzt für die Gründung des European Molecular Biology Laboratory (EMBL), dessen Gründungsdirektor er wurde, und hat Wissenschaftspolitik und *government advising* auf sehr hohem Niveau gemacht. Das war eine *trajectory*, die für meine Geschichte sehr wichtig ist.

Anfänglich wollte Kendrew mir nur Zugang zu den Papieren geben, die mit seiner Proteinforschung zu tun hatten. Es brauchte ziemlich viel Überredung, um ihm zu erklären, dass die anderen Sachen nicht irrelevant waren für mein Projekt. Er hatte drei Kategorien, mit denen er meine Wunschliste von Papieren, die ich einsehen wollte, markierte: *personal*, da konnte man noch nicht ran, *irrelevant for your project* und Sachen, die ich einsehen durfte. Und *irrelevant for your project* waren ziemlich viele Sachen, zum Beispiel auch alle wissenschaftspolitischen Unterlagen. Zu denen hat er mir nur langsam Zugriff gewäht, aber am Ende hatte ich Zugang zu allem, was ich wollte. Ich glaube, über dieses große Archiv und die Gespräche mit Kendrew und dann auch mit anderen Leuten, wie zum Beispiel Sydney Brenner,^58^ habe ich meine Ideen entwickelt, wie ich diese Geschichte anpacken würde.

**AtH**: Letztlich hast Du aus dem Beirat Deines Projektes heraus Deine Zeitgeschichte der Wissenschaft entwickelt, oder? Ich finde, dass das unter anderem über Kendrew gelaufen ist, sehr interessant und auch, dass er meinte, Dich auf sich selbst hinweisen zu müssen …

**SdC**: Ich hatte ja schon angefangen mit dem Projekt, da traf ich ihn nach einer Weile. Ich hatte sehr stark das Gefühl, dass ich mich zunächst selbst orientieren müsste und mich nicht gleich von irgendwelchen Geschichten, die mir Leute erzählen, leiten lassen wollte. Später, aber *viel* später erst, ich würde sagen nach fünf Jahren, wurde ich von Richard Henderson,^59^ dem damaligen Direktor vom Laboratory of Molecular Biology in Cambridge, über das ich arbeitete, kontaktiert. Ich glaube, die Finanzierung der Wellcome‐Stelle lief aus, und dann – mit einigen anderen Zwischenaufenthalten – kam eben nochmal dieses Angebot vom LMB, die suchten jemanden, der die Geschichte des LMB schreibt, und sind auf mich gekommen. Sie haben mich sehr großzügig finanziert und nochmal umfassenderen Zugang und Mitarbeit vom LMB zugesichert. Also, wie gesagt, ich war schon sehr aktiv dabei, das Buch war bereits sehr klar strukturiert, als das LMB auf mich zukam. Da hat Henderson ziemlich schnell entschieden, dass das Labor sowohl mein Buch weiter unterstützen würde, als auch eben – *I think, they called it a family history* – fördern würde, wofür sie John Finch^60^ vom LMB gewannen. Wir haben darüber geredet, was mein Buch ist, was es leisten kann und was es nicht abdecken kann. Und dann kam Henderson zur Entscheidung, beide Projekte zu fördern, und ich fand das sehr gut, es hat mir völlige Freiheit gelassen. Gleichzeitig haben sie dann mit Finchs Buch bekommen, was sie eigentlich eher wollten oder erwarteten.^61^ Übrigens, obwohl die Entscheidung gemeinsam gefallen ist, kam Finchs Buch erst fünf oder sechs Jahre nach meinem heraus, viel später, weil er noch aktiv im Labor war und das Buch zusammenzustellen doch viel mehr Arbeit war, als er anfänglich dachte. Erst als Emeritus hat er sich darauf konzentrieren können. Wir haben unsere Manuskripte ausgetauscht und gegenseitig kommentiert. Es war ganz klar, dass mein Buch für die Wissenschaftsgeschichte geschrieben ist, aber hoffentlich auch für das Labor interessant sein würde. Und so war es dann auch. Ich glaube, das hatte ich alles Henderson zu verdanken, der wirklich an der Geschichte interessiert war, der auch das Archiv am LMB aufgebaut hatte und Annette Faux als Archivarin einstellte. Sie vertrat ihre Stelle mit viel Elan und stand immer als Ansprechpartnerin zur Verfügung. Leider ist sie letztes Jahr frühzeitig verstorben, doch das Archiv wird weiter bestehen.

**MG**: Das Interview als Methode der wissenschaftshistorischen Forschung hast Du auch in diesem Kontext für Dich entwickelt und angewandt?

**SdC**: Das begann mit den Gesprächen mit Kendrew. Da wurde klar, dass ich Oral History als Methode benutzen würde. Dafür bot der Wellcome Trust einen Trainingskurs an, in den ich mich einschrieb. Es war, wenn ich das richtig erinnere, ein drei Tage dauernder, intensiver Kurs an der University of Essex, wo man in die Methoden und Probleme der Oral History eingeführt wurde. Was ich für meine Forschung gemacht habe, waren allerdings nicht Oral History‐Interviews im strengen Sinne: Wir lernten bei dem Kurs, dass wir Nicht‐Personen sein müssten, möglichst neutral im Auftreten, Kleidung etc. Der *interviewee* müsste reden, die Lebensgeschichte erzählen, und das ist ja nicht das, was ich gemacht habe. Ich habe – *I called it research‐interviews* gemacht, mit viel gezielteren Fragen. Natürlich stellte ich Fragen so, dass sie nicht *binding* waren, aber trotzdem viel gezielter, auch mit der Möglichkeit zurückzufragen. Meine Interviews entsprachen überhaupt nicht dem, wie ich es gelernt hatte. Aber die Kurs‐Erfahrung war trotzdem sehr wichtig als Einführung in diese Thematik und in allen Aspekten des Interviewens, von technischen Fragen bis zu legalen und ethischen. Dann habe ich diesen Aufsatz geschrieben über den Gebrauch von Interviews in der Wissenschaftsgeschichte, der aus der Auseinandersetzung mit meinen wissenschaftshistorischen Kollegen entstand.^62^ Sie sahen Interviews als eine soziologische Arbeitsmethode, der sie sehr skeptisch gegenüberstanden. Im Allgemeinen fanden die Wissenschaftshistoriker, mit denen ich damals gesprochen habe, es eine sehr erschreckende Vorstellung, dass die Akteure mitreden dürften, lebendig sind und zurückreden können [lacht]. Aber es gibt natürlich so etwas wie Gegenwartsgeschichte, wo diese Möglichkeit dazugehört.

Damals war das HPS‐Department in Cambridge noch sehr stark an *Victorian Science* orientiert, die meisten Leute haben zum 19. Jahrhundert gearbeitet und es war wirklich relativ ungewöhnlich, sich um das 20. Jahrhundert und dann auch noch das späte 20. Jahrhundert zu kümmern. Eine Rolle spielte natürlich die *thirty‐year‐rule* im Archiv, die ein ernsthaftes Problem darstellt. Daher auch der Rückgriff auf Interviews. Und dann natürlich die Frage: Kann man so Geschichte machen, macht das Sinn, ist man nicht zu nahe dran … Das hat mich motiviert, diesen Aufsatz zu schreiben und mich auch sonst manchmal über Fragen des Archivs zu äußern, weil wenn man rezente Wissenschaftsgeschichte betreibt, dann arbeitet man nahe an der Zeit, in der die Archive entstehen. Oft ist man in diesem Prozess auch direkt miteinbezogen.^63^

**AtH**: Würdest Du sagen, dass Du bei den Gesprächen, die Du schließlich geführt hast, auf der einen Seite direkt gefragt hast, weil Du bestimmte Dinge einfach wissen musstest oder wissen wolltest, als eine Art von Informationsbeschaffung, auf einer ganz pragmatischen Ebene? Welche Rolle haben dann auf der anderen Seite Deine wissenschaftstheoretischen, Deine epistemologischen Überlegungen, die Du ja nicht abstellen konntest, dabei gespielt? Hast Du nach bestimmten Bereichen gefragt oder hast Du vor dem Hintergrund bestimmter epistemologischer Fragen auch Deinen Fragenkatalog entwickelt?

**SdC**: Die Fragen nach der Methode spielten in den Interviews selber nicht so eine Rolle. Welche Fragen ich gestellt habe, hing mehr mit der Biografie dieser Leute zusammen … Zum Beispiel Kendrew, der dieses umfangreiche Archiv hatte, war sehr einflussreich gerade auch in Bezug auf die wissenschaftliche, institutionelle und wissenschaftspolitischen Definition und Verankerung des neuen Forschungsfelds. Im Allgemeinen interessierten mich die Leute, die in der Anfangsphase dabei waren und eben auch noch erreichbar waren, in Cambridge oder anderswo. Ich habe mich dann ziemlich stark mit Proteinforschung auseinandergesetzt, dem *early use of computers*, *postwar science policy*, das waren dann so Themen, die mich interessierten. Eine andere Person war sehr wichtig für mich, Michael Fuller.^64^ Er hat ganz jung als Laboraushilfe begonnen, ich glaube mit 17 Jahren, und ist bis zum Ende seiner Karriere im LMB geblieben. Er hat anfänglich ganz praktische Sachen im Labor gemacht.

**MG**: Das sogenannte „Mädchen für alles“.

**SdC**: „Mädchen für alles“, so fing er an, und hat sich dann langsam zum *laboratory manager* hochgearbeitet. Er personifizierte die Institution, die er hatte wachsen sehen und zu deren Erfolg er seinen Beitrag geleistet hatte. Er hat mich herumgeführt, daran erinnere ich mich. Er hat mir alles gezeigt und mir geraten, mit wem ich reden sollte, wer schon lange da war, wer wichtig war, wer ein gutes Gedächtnis hat. So habe ich angefangen, mir einen Plan zu machen, mit wem ich reden wollte. Mit den *Nobel Prize winners*, ja, aber auch mit den Mechanikern und Technikern, die viele der Geräte gebaut oder bedient hatten und nahe an der Praxis des Experimentierens waren. Es war auch ein bisschen ein *snowball*‐System, jeder hat mir gesagt, mit wem ich noch unbedingt reden müsse und so weiter und so weiter … Oral History war ja sehr stark von der Idee getragen, dass es ein *tool* sei, um eben denjenigen eine Stimme zu geben, die sonst in der Geschichte keine hatten. Man musste eigentlich mehr rechtfertigen, warum man die Spitzenwissenschaftler interviewte, von denen es ja im Labor sehr viele gab und die oft schon über ihre Arbeiten und Erfahrungen geredet und geschrieben hatten. Aber natürlich kann man sie nicht einfach übergehen, denn man muss verstehen, wie sie zu ihren Positionen gekommen sind. Trotzdem ist es klarerweise etwas anderes, wenn man jemanden interviewt, der schon oft öffentlich geredet hat, der schon sozusagen seine Geschichte parat hat, oder jemanden, der eigentlich denkt, er hat nichts zu erzählen. Da muss man verschiedene Strategien anwenden.

**AtH**: Erzähle uns bitte noch kurz etwas über das Verhältnis von Wissenschaftsgeschichte und Wissenschaftsphilosophie, gerade, weil Du eine derjenigen Wissenschaftshistorikerinnen bist, für die dadurch, dass sie sehr stark in der Zeitgeschichte gearbeitet haben, vielleicht auch das Verhältnis von Geschichte und Philosophie in den Hintergrund getreten ist?

**SdC**: Meinst Du, wie diese Frage allgemein diskutiert wurde oder speziell in Bezug zu meiner Arbeit?

**MG**: Zwei Aspekte scheinen mir besonders relevant: Zum einen, dass Du einen Weg über die kontinentale Philosophie in Deutschland und dann über diesen experimentellen Raum West‐Berlin, den Kontext der Historischen Epistemologie und dann in die angelsächsische *History and Philosophy of Science* (HPS) gegangen bist. Wie konntest Du diese verschiedenen Ansätze für Dich produktiv machen? Zum anderen, vielleicht ein bisschen spekulativer, wo steht die Auseinandersetzung zwischen HPS und Historischer Epistemologie jetzt, welche Herausforderungen gibt es?

**SdC**: HPS hieß und heißt das Department in Cambridge, *History and Philosophy of Science*, aber meine Wahrnehmung damals war, dass die Philosophie und die Geschichte ziemlich nebeneinander herliefen. Einige Leute haben es verbunden, Nick Jardine^65^ zum Beispiel, aber der war ja auch nicht klar zuzuordnen. Er hat einen sehr interessanten intellektuellen Lebenslauf. Und natürlich auch die Gründer des Departments, Gerd Buchdahl^66^ und Mary Hesse,^67^ sie hatten eine Vision, wie Geschichte und Philosophie zusammenhängen, aber die waren beide schon emeritiert, als ich nach Cambridge kam. Doch zum Beispiel die Geschichte der Physik, wie sie von Schaffer vertreten wurde, und die Philosophie der Physik oder Quantenmechanik, wie sie von Michael Redhead^68^ betrieben wurde, hatten meiner Ansicht nach nicht viele Berührungspunkte. Aber beide hatten viele Schüler, und Physik war auf jeden Fall das dominierende Thema zu der Zeit. Das hat sich später geändert. Aber auch was später unter Philosophie der Biologie lief, war sehr stark analytisch orientiert, wie Du ja schon angedeutet hast, Mathias. Da gab es für mich nicht viele Ansatzpunkte. Auch musste ich ja erstmal einen Griff auf die Geschichte der Molekularbiologie bekommen.

Ich glaube, dass mein Interesse an theoretischen Fragen sich im Interesse an Historiographie niedergeschlagen hat, also, wie schreibt man Geschichte. Dahinter steht auch die Frage, was ist Wissenschaft, denn je nachdem, wie man Wissenschaft versteht, schreibt man ihre Geschichte. Für solche Fragen waren diese neuen Ansätze in der Wissenschaftsgeschichte, vom *strong program* zum Konstruktivismus und so weiter durchaus geeignet, weil dort ja solche Fragen verhandelt wurden. Und es gab Bezüge zu Kuhn, zu Wittgenstein – das waren Dinge, die hat man diskutiert. Da war ich immer froh, dass ich Wittgenstein in der Philosophie in Konstanz ausführlich gelesen habe, dass ich diesen philosophischen *background* hatte, dass ich wusste, wenn man Kant oder Metaphysik oder auch Epistemologie und Ontologie sagt, was ich mir darunter vorstellen sollte. Aber irgendwie habe ich mich nicht mehr aktiv damit auseinandergesetzt, mit Philosophie und ihrer Schnittstelle zur Geschichte. Ich habe nochmal einen Aufsatz geschrieben, und zwar war das für die Festschrift für Wellmer, meinen Doktorvater, die einige seiner Schüler herausgegeben haben. Der Band hieß *Zur Verteidigung der Vernunft gegen ihre Liebhaber und Verächter.* Da habe ich versucht, diese neuen wissenschaftshistorischen Ansätze nochmal philosophisch zu befragen.^69^ Ich habe mich auf Ian Hacking und Joseph Rouse, auch Merleau‐Ponty und andere berufen. Da habe ich mich nochmal richtig hineinbewegt, auch in ein paar anderen kleinen Aufsätzen, aber ich habe mich nicht mehr wirklich mit Wissenschaftsphilosophie in der traditionellen Art auseinandergesetzt.

Ich würde sagen, die Philosophie der Biologie ist wirklich explodiert. Sie handelt nicht mehr nur von Evolutionsbiologie (und auch da haben sich die Fragen sehr verändert), sondern hat ihre Fragenpalette weit geöffnet, ich weiß nicht, Fragen über Modelle, Modellorganismen, Repräsentation, Daten, mechanistische Erklärungen und Prozesse. Das habe ich mitbekommen, einiges davon auch gelesen, einiges davon auch benutzt in meinen Arbeiten, aber nie richtig teilgenommen an den Diskussionen, obwohl einige meiner Aufsätze von Wissenschaftsphilosophen durchaus rezipiert worden sind. Ich würde sagen, auch die Wissenschaftsphilosophie hat irgendwann diesen Praxis‐Turn mitgemacht und sich interessiert für das, was die Wissenschaften eben machen, wie Husserl schon gesagt hat. Da haben sich sehr viele Sachen verändert, aber ich meine, es ist auch eine Frage, was man noch zeitlich hinkriegt, nicht wahr? Es gibt jetzt von Seiten der Feministischen Wissenschaftstheorie und der *environmental humanities* ein neues Interesse an Merleau‐Ponty. Sogar in Cambridge gab es kürzlich ein *reading*‐Seminar über Merleau‐Ponty, das würde mich interessieren, wie sie Merleau‐Ponty jetzt lesen. Oder auch in den *cognitive sciences*, wofür ich mich auch ein bisschen interessiert habe, da die Entwicklung zu sehen. Auch diese Fragen kamen aus der Beschäftigung mit Merleau‐Ponty, die Frage von *embodiment* in *artificial intelligence* und so weiter. Das sind Fragen, die würden mich schon interessieren, aber jetzt ist es eine Frage der Zeit, wofür und mit was man sich beschäftigen kann in den Arbeitskreisen, in die man eingebettet ist.

**AtH**: Ein gutes Schlusswort: Man kann nicht alles machen.

**MG**: Das stimmt, aber eine Frage möchten wir Dir unbedingt noch stellen, und zwar zu Familie und Beruf. Du hast von sehr verschiedenen Stationen, von einem extrem dynamischen Leben berichtet, in dem auch nicht immer vorab klar war, wohin die nächste Station führt, und wir haben auch über Finanzierung geredet und die Unsicherheiten, die das mit sich bringt. Was würdest Du jüngeren Leuten heute mit auf den Weg geben? Was sind die wichtigen Erfahrungen für Dich gewesen?

**SdC**: Das ist schwierig, weil ich jetzt im amerikanischen System bin. Ich finde wirklich, dass wir kostenlos studieren konnten, macht den grundsätzlichen Unterschied aus zu dem System, in dem ich jetzt bin. Ich meine, für uns war doch ein Jahr mehr studieren eher attraktiv. Ich hatte immer Stipendien oder habe gearbeitet und es war auch lange prekär, aber irgendwie war es eine Prekarität, die trotzdem lebbar war, weil man zum Beispiel keine Studiengebühren zahlen musste, um zu promovieren. Und weil sich keine Schulden angehäuft haben, wie bei den Studierenden hier in den USA. Das ist wirklich ein Grundunterschied, der die ganze *attitude* zum Studium verändert, das darf man nicht aus dem Blick verlieren. Das gilt übrigens auch für meine jetzige Universität, die UCLA, denn wir hatten früher hier einige Doktoranden, die kein volles finanzielles Paket hatten. Sie mussten sich eben durch *assistantships* und so weiter durchschlagen. Das ging aber, weil die Studiengebühren an der UCLA noch nicht so hoch waren. Seitdem die Studiengebühren erheblich gestiegen sind – auch hier, wobei sie immer noch niedriger sind als an anderen Unis –, geht das nicht mehr. Man kriegt das nicht mehr hin, man kann nicht einmal die Studiengebühren verdienen, und dann hat man noch nicht gelebt. Wir akzeptieren jetzt nur noch Promovierende, denen wir ein volles finanzielles Paket anbieten können, was aber auch heißt, dass wir weniger Bewerber:innen zulassen können.

Ich war sehr lange auf befristeten Verträgen. Es waren sehr gute Verträge, sie waren langfristig, bis zu fünf Jahren, und ich konnte immer meine eigenen Projekte verfolgen, musste nicht irgendwelche Auftragsarbeiten machen oder mich in neue Projekte einarbeiten. Das ist auch nicht so einfach, glaube ich, heutzutage, obwohl die *soft money*‐Förderung viel verbreiteter ist, als sie es noch vor zehn, zwanzig Jahren war. Aber die Tatsache, dass ich auf Forschungsverträgen war, hat mir mehr Flexibilität gegeben, auch im Hinblick auf Familie. Es hat mir weniger klare Freizeiten gegeben, aber ich glaube, dass es mir trotzdem geholfen hat, Familie und Beruf integrieren zu können – obwohl ich das sicherlich nicht als Modell vorschlagen will. Zudem war es lokal sehr verschieden: In Berlin, zum Beispiel, war die Kinderbetreuung viel besser als in Süddeutschland, da es viel normaler war, die Kinder in der Kita zu haben bis drei, vier oder fünf Uhr, was in Süddeutschland noch überhaupt nicht der Fall war. Also die Jahre in Berlin waren in der Hinsicht einfacher. Ich war da vorgeprägt von einem Jahr in Amerika, wo unsere einjährige Tochter auch schon in die Kita ging und es liebte. In England fängt die Schule schon mit vier an, auch das hat geholfen. Netzwerke waren sehr wichtig: ein unterstützender Partner, kollaborative Kinder. Irgendwie ging es.

**MG**: A propos Befristung: Das ist ein interessanter, fast diabolischer Beitrag zur aktuellen deutschen Debattenlage.^70^

**SdC**: Es war nicht die Befristung, sondern die Tatsache, dass ich auf Forschungsstellen war mit weniger festen Verpflichtungen. Aber ich habe Schwierigkeiten das den jungen Leuten zu vermitteln. Ich glaube, ich bin an einem bestimmten Punkt ein Vorbild dafür geworden, wie man eben sozusagen „Karriere machen“ und Kinder haben kann, aber wenn ich das sehe bei den Doktoranden, die jetzt auf dem *job market* sind, die halten die Unsicherheit nicht mehr aus. Es ist auch eine Frage der Zeit, denke ich. Unsichere Zeiten, in denen man mehr Sicherheiten braucht. Ich weiß nicht, warum ich so lange gedacht habe, wenn es in dem Moment irgendwie stimmte, dann war es gut genug. Ein bisschen davon ist sicherlich biographisch, aber ich glaube schon, dass es einfach andere Zeiten waren. Nicht, dass wir dachten, es gäbe all diese Stellen für uns, das ist mir nie so nahegelegt worden, in der Biologie nicht, wo uns alle abgeraten haben, in die Forschung zu gehen: „Frauen und Diplom‐Biologie? Was wollt ihr damit?“ Das wäre doch nicht kompatibel mit Kinderkriegen, wurde uns explizit gesagt – zu einem Zeitpunkt, an dem ich noch überhaupt nicht an Kinder dachte. In der Philosophie, da wurde mir nahegelegt, dass die Berufschancen in den Naturwissenschaften viel besser seien, eine Dissertation in der Philosophie also nur Sinn machte, wenn ich plante, in die Naturwissenschaft zurückzugehen. Nie wurde mir die Idee vermittelt, dass man in der Philosophie einfach eine Karriere machen konnte, zumal mit Kindern. In der Wissenschaftsgeschichte sowieso nicht.

Aber für mich wäre es eher beängstigend gewesen, eine feste Stelle zu haben, glaube ich*, early on*. Ich war noch auf der Suche.

## Footnotes

1 Carsten Bresch (1921–2020), Genetiker, Albert‐Ludwigs‐Universität.

2 Max Henning Ludwig Delbrück (1906–1981), Genetiker und Biophysiker, Californian Institute of Technology (Caltech).

3
Monod 1970.

4
Bresch 1964. Ab der zweiten Auflage von 1970 war Rudolf Hausmann Koautor dieses Werkes.

5 Soraya de Chadarevian et al. 1979.

6 Rainer Hertel (*1937), Molekularbiologe, Albert‐Ludwigs‐Universität Freiburg.

7
de Chadarevian 1996.

8 Jean Piaget (1896–1980), Begründer der genetischen Epistemologie, Universität Genf.

9 Claude Lévi‐Strauss (1908–2009), Ethnologe, Collège de France, Paris.

10 Lorraine Daston (*1951), Wissenschaftshistorikerin, Max‐Planck‐Institut für Wissenschaftsgeschichte (MPIWG), Berlin.

11 Catherine Wilson (*1951), Philosophin, University of York.

12 Christiane Schildknecht (*1958), Philosophin, Universität Luzern.

13
Öko‐Institut Freiburg 1984.

14 Albrecht Wellmer (1933–2018), Philosoph, Freie Universität Berlin.

15 Jürgen Mittelstraß (*1936), Philosoph, Universität Konstanz.

16
de Chadarevian 1990.

17 Wolf Lepenies (*1941), Soziologe, Freie Universität Berlin.

18 Timothy Lenoir (*1948), Wissenschaftshistoriker, University of California, Davis.

19 Simon Schaffer (*1955), Wissenschaftshistoriker, University of Cambridge.

20 Thomas P. Hughes (1923–2014), Technikhistoriker, University of Pennsylvania.

21 Yehuda Elkana (1934–2012), Wissenschaftstheoretiker und Wissenschaftshistoriker, u.a. Wiko und MPIWG, Berlin.

22 Christoph Meinel (*1949), Wissenschaftshistoriker, Universität Regensburg.

23 Michael Becker (*1944), Wissenschaftskoordinator, Verbund für Wissenschaftsgeschichte und Wiko, Berlin.

24 Herbert Mehrtens (1946–2021), Wissenschaftshistoriker, TU Berlin.

25 Rolf Winau (1937–2006), Medizinhistoriker, Freie Universität Berlin.

26 Hans‐Peter Schmiedebach (*1952), Medizinhistoriker, Universitätsklinikum Hamburg.

27 Michael Hubenstorf (*1954), Medizinhistoriker, Universität Wien.

28 Volker Hess (*1962), Medizinhistoriker, Charité Berlin.

29 Hans‐Jörg Rheinberger (*1946), Wissenschaftshistoriker, MPIWG, Berlin.

30 Nicolaus Sombart (1923–2008), Kultursoziologe, Wiko und FU Berlin.

31 Peter McLaughlin (*1951), Wissenschaftstheoretiker und ‐historiker, Universität Heidelberg. Siehe dazu Grote et al. 2021.

32
Wolfgang Lefèvre (*1941), Wissenschaftshistoriker, MPIWG, Berlin. Siehe dazu Grote et al. 2022.

33 Ursula Klein (*1951), Wissenschaftshistorikerin, MPIWG, Berlin.

34 Jürgen Renn (*1956), Wissenschaftshistoriker, MPIWG, Berlin.

35
Lepenies 1991.

36 Aus einzelnen Klassen der Akademie der Wissenschaften der DDR gingen nach 1990 sogenannte von der Max‐Planck‐Gesellschaft getragene „Forschungsschwerpunkte“ hervor, die nach Begutachtung übernommen werden konnten. Der bereits erwähnte Forschungsschwerpunkt Wissenschaftsgeschichte und das dann 1994 neu gegründete Max‐Planck‐Institut für Wissenschaftsgeschichte hängen also – gemeinsam mit dem hier geschilderten Verbund für Wissenschaftsgeschichte – eng zusammen. Siehe dazu auch Ash 2023, S. 88–89, 117–118.

37 Peter Galison (*1955), Wissenschaftshistoriker, Harvard University.

38 Nancy Cartwright (*1944), Wissenschaftstheoretikerin, Durham University und University of California, San Diego.

39 Robert E. Kohler (*1937), Wissenschaftshistoriker, University of Pennsylvania.

40 Andrew Warwick, Wissenschaftshistoriker, Imperial College London.

41
Rheinberger 1992.

42 Skúli Sigurdsson (*1958), Wissenschaftshistoriker, Berlin.

43 Sybilla Nikolow (*1963), Wissenschaftshistorikerin, Universität Bielefeld.

44 Robert Brain, Wissenschaftshistoriker, University of British Columbia.

45 William Clark (1953–2017), Wissenschaftshistoriker, University of California, Los Angeles.

46 Michael Hagner (*1960), Wissenschaftshistoriker, ETH Zürich.

47 Bettina Wahrig (*1956), Wissenschaftshistorikerin, TU Braunschweig.

48
Hagner und Wahrig‐Schmidt 1992.

49 Tore Frängsmyr (1938–2017), Wissenschaftshistoriker, Uppsala Universität.

50 John Heilbron (1934–2023), Wissenschaftshistoriker, University of California, Berkeley.

51 Giuliano Pancaldi (*1946), Wissenschaftshistoriker, Universität Bologna.

52 Dominique Pestre (*1950), Wissenschaftshistoriker, École des hautes études en sciences sociales (EHESS), Paris.

53 Frederic L. Holmes (1932–2003), Wissenschaftshistoriker, Yale University.

54 Renato G. Mazzolini (*1945), Wissenschaftshistoriker, Universität Trento.

55
de Chadarevian 1993b (durchgesehene deutsche Übersetzung: de Chadarevian 1994).

56 De Chadarevian 2002.

57 John Kendrew (1917–1997), Biochemiker und Kristallograph, MRC Laboratory of Molecular Biology Cambridge und European Molecular Biology Laboratory, Heidelberg.

58 Sydney Brenner (1927–2019), Molekulargenetiker, MRC Laboratory of Molecular Biology, Cambridge. Später an zahlreichen anderen Institutionen in den USA und in Asien tätig.

59 Richard Henderson (*1945), Biophysiker, MRC Laboratory of Molecular Biology, Cambridge.

60
John Finch (1930–2017), Molekularbiologe, MRC Laboratory of Molecular Biology, Cambridge.

61
Finch 2008.

62
de Chadarevian 1997; siehe zu *Oral History* auch Keuck und de Chadarevian 2025.

63 Siehe z.B. de Chadarevian 2016.

64 Michael Fuller (1936–2019), Laboratory Services Manager und Laboratory Steward, MRC Laboratory of Molecular Biology, Cambridge.

65 Nicholas Jardine (*1943), Mathematiker, Wissenschaftshistoriker und Philosoph, University of Cambridge.

66 Gerd Buchdahl (1914–2001), Wissenschaftsphilosoph, University of Cambridge.

67 Mary Hesse (1924–2016), Wissenschaftsphilosophin, University of Cambridge.

68 Michael Redhead (1929–2020), Philosoph der Physik, University of Cambridge.

69
de Chadarevian 1993a.

70 N.B.: Zu der beschriebenen Zeit existierte kein Wissenschaftszeitvertragsgesetz.
